# Inauguration of the Jenner Monument

**Published:** 1858-07

**Authors:** 


*Inauguration of the Jenner Monument.*—On Monday, May 18, the Jenner monument, recently erected in Trafalgar Square, London, was inaugurated by Prince Albert. The monument consists of a bronze statue, representing the great discoverer in a sitting posture, and is placed next to the statue of Sir Charles Napier, at the west side of the square. Contributions to the fund for its erection have been received from all parts of the world. The statue was uncovered during the day, and at three o'clock in the afternoon, Prince Albert arrived at the Royal College of Physicians, in Pall Mall East, where it had been arranged that the inauguration should take place. Amongst the company present, were the Marquis of Lansdowne, Sir John Forbes, Sir J. Macgregor, Bart., Professor Ferguson, Drs. Elliotson, Hawkins, Paget, Conolly, Sir Charles Landseer, R. A., Mr. D. Maclise, R. A., &c. His Royal Highness said that he attended there to do honor, with those present, to the memory of Jenner, that being the anniversary of Jenner's birthday, and in order to mark his sense of the inestimable benefits bestowed upon the human race by that great philosopher and philanthropist. The discovery of vaccination was not the result of mere accident like many other discoveries, but it was the result of long and thoughtful observation and reflection, to which the discoverer's whole life was devoted. This country might be justly proud to number among her sons such a man as Jenner, for no man had been able to save so many lives as he had been enabled to do. (Loud applause.) His contemporaries had testified their approbation and feeling of gratitude for the important public service he had rendered, but it was reserved for them that day to inaugurate a memorial as a mark of their appreciation of Jenner's services in the cause of humanity. (Applause.) He hoped that statue would be long preserved to give the features of this benefactor of humanity for the contemplation and admiration of generations to come. (Great applause.) In replying to a vote of thanks, His Royal Highness expressed a hope that vaccination would be still further spread, for it was deplorable to think that through neglecting it, there were still in this country about 5000 individuals annually numbered among the victims.

The above extract gives an account of the inauguration of the Jenner monument, which has been so long in contemplation, and for which the profession of the United States have contributed much more than that of any other nation. The reward of the statesman, and the soldier, is seldom forgotten, and their names are on every tongue; while the great physician often labors a long life unnoticed and unknown except by his co-workers, and the names most revered by the profession are often unheard of by others.

Honor to the men that do honor to the memory of the great discoverer of vaccination, a discovery which was the result of such weary years of toil and persecution, and which has made a disease which was formerly the scourge of humanity, almost a scientific curiosity.

---

*Palmer's Artificial Leg.*—Mechanical ingenuity cannot be turned to a better purpose than the supplying of lost members, to those who are so unfortunate as to be mutilated by the surgeon's knife. Messrs. Palmer & Co. have brought to such a degree of perfection the manufacture of artificial limbs, that persons who have lost even both legs, are enabled to trudge about with the rest of mankind, nearly as well, on ordinary occasions, as the best of them.

The legs of Mr. Palmer are now admitted to be equal, if not superior to any in the world, and his skill in adapting them to every peculiarity of the case, adds greatly to their usefulness. The references which Mr. Palmer publishes by permission, number some of the most distinguished European and American surgeons, and we can cheerfully add our recommendation to this triumph of American ingenuity.

---

*Emerson's Magazine and Putnam's Monthly.*—We have been compelled to defer our notice of this valuable periodical, in consequence of the press of matter, but now perhaps can more earnestly testify to its merits, as it has been our welcome visitor for some months. Doctors should not be mere doctors, and it was not intended that the human mind should always be occupied with the severe studies of a profession. From the pages of the literary journals of the day, among which this magazine holds a high rank, one can derive, not only information which is necessary to every one, be his occupation what it may, but a pleasure which severe professional duties eminently prepare the mind to enjoy.

The magazine may be obtained of Messrs. Oaksmith & Co., 112 and 114 William street, New York, at $3 a year, who offer as an inducement to their subscribers, a magnificent steel plate engraving, which for the seventh volume, commencing July, 1858, is the "Last Supper."

---

*The Maine Medical and Surgical Reporter.* Conducted by W. R. RICHARDSON, M. D., and R. W. CUMMINGS, M. D.

This is the first number of a new monthly Journal of 48 pages, published at Portland, Maine. It presents a good appearance, and we welcome it as an interesting addition to our exchange list. The price is $3 per annum.

---

*Death of Prof. Hare.*—We learn with deep regret, of the death of Prof. Hare, of Philadelphia, who was long the Prof. of Chemistry in the venerable University of Penn. Prof. Hare's contributions to chemical science have won for him a lasting reputation; he has justly been numbered among our great men.

*Plates illustrative of Wilson on Diseases of the Skin.* Philadelphia: BLANCHARD & LEA. 1857.

The admirable treatise which these plates are intended to illustrate, is now the standard work on diseases of the skin, in the English language, and is rendered entirely complete by the faithful and graphic plates which have lately been issued. The only means authors have of removing some of the difficulties which attend the study of cutaneous diseases, is by a faithful delineation of their actual appearance. We have already given testimony as to the excellence of the treatise, and can only say that the diseases of the skin are as faithfully illustrated in the one, as they are described in the other.

---

From the North American Medico-Chirurgical Review.

*Medical Students in the United States during the Session of* 1857–'58.—We publish a list of the students and graduates of the different schools, which, although reliable, must necessarily be imperfect from the fact of our not being in possession of all the catalogues and circulars, anxious as we have been to obtain them:

| | Students. | Graduates. |
|---|---|---|
| Jefferson Medical College, | 501 | 209 |
| University of Pennsylvania, | 437 | 145 |
| University of New York, | — | 127 |
| University of Nashville, | 358 | 109 |
| University of Lousiana, | 276 | 68 |
| Georgia Medical College, | — | 61 |
| College of Physicians and Surgeons, | — | 53 |
| St. Louis Medical College, | 125 | 49 |
| Medical College of Ohio, | — | 43 |
| Rush Medical College, | 100 | 36 |
| Pennsylvania Medical College, | 140 | 35 |
| New Orleans School of Medicine, | 126 | 33 |
| New York Medical College, | — | 33 |
| University of Louisville, | — | 31 |
| University Medical College, | — | 27 |
| Missouri Medical College, | — | 25 |
| Memphis Medical College, | — | 19 |
| Philadelphia College of Medicine, | 63 | 18 |
| Massachusetts Medical College, | — | 16 |
| Transylvania University, | — | 12 |
| Oglethorpe Medical College, | 37 | 11 |
| Kentucky School of Medicine, | — | 11 |
| Starling Medical College, | — | 10 |
| University of Buffalo, | — | 9 |
| Medical Department of Yale College, | — | 6 |
| Medical College of South Carolina, | 276 | — |
| Richmond Medical College, | 50 | — |
| University of Virginia, | 88 | — |

